# Don’t panic? Or don’t conflate?

**DOI:** 10.1093/hropen/hoag061

**Published:** 2026-06-27

**Authors:** Norbert Gleicher, Sonia Gayete-Lafuente, Lara Guijarro-Baude, Pasquale Patrizio, David F Albertini, David H Barad

**Affiliations:** The Center for Human Reproduction, New York, NY, USA; The Foundation for Reproductive Medicine, New York, NY, USA; Laboratory of Synthetic Embryology, The Rockefeller University, New York, NY, USA; Department of Obstetrics and Gynecology, Medical University of Vienna, Vienna, Austria; The Center for Human Reproduction, New York, NY, USA; The Foundation for Reproductive Medicine, New York, NY, USA; The Center for Human Reproduction, New York, NY, USA; Department of Obstetrics and Gynecology, Hospital Universitari de Vic, Barcelona, Spain; The Center for Human Reproduction, New York, NY, USA; Division Reproductive Endocrinology & Infertility, Miller School of Medicine, University of Miami, Miami, FL, USA; The Center for Human Reproduction, New York, NY, USA; Bedford Research Foundation, Boston, MA, USA; The Center for Human Reproduction, New York, NY, USA; The Foundation for Reproductive Medicine, New York, NY, USA

Dear Editor,

We appreciate the Letter to the Editor by Hill *et al*. (2026), regarding our recent *Human Reproduction Open* publication on declining IVF treatment efficiency in the USA (Gleicher *et al*., 2026). Their Letter raises several important points. However, we believe it also highlights a central conceptual problem in interpreting contemporary IVF outcome reporting: the conflation of true fertility preservation with the much broader category of treatment-related embryo cryopreservation.

Hill *et al*. (2026) repeatedly frame embryo banking primarily as a fertility-preservation strategy. Yet, the overwhelming majority of frozen IVF cycles in contemporary US practice are unlikely to represent true long-term fertility preservation. Instead, most reflect routine management strategies associated with modern IVF practice, including preimplantation genetic testing for aneuploidy (PGT-A), systematic freeze-all protocols, deferred embryo transfer, elevated progesterone levels, ovarian hyperstimulation syndrome prevention, blastocyst biopsy logistics, endometrial synchronization, and embryo accumulation strategies in poor-prognosis patients.

These treatment-related freeze cycles are fundamentally different from fertility preservation for elective or medical indications. Our article explicitly acknowledged that embryo banking may represent ‘valid medical or patient-driven decisions, such as fertility preservation or pre-treatment storage’. At the same time, we noted that the magnitude of the observed increase strongly suggests widespread use in non-medically indicated settings. Indeed, the 902% increase in embryo banking cycles between 2012 and 2021 temporally parallels the widespread adoption of vitrification, blastocyst biopsy, and especially PGT-A-associated freeze-all strategies far more closely than it parallels the later rise in elective fertility preservation.

Importantly, true fertility preservation likely represents only a minority of all-freeze cycles nationally. Recent Society for Assisted Reproductive Technology (SART) reports themselves increasingly distinguish fertility-preservation cycles from routine IVF treatment cycles, implicitly recognizing this important distinction. The Letter from Hill *et al*. (2026), however, appears to merge these fundamentally different clinical scenarios into a single category of ‘embryo banking’, thereby obscuring the degree to which routine IVF practice has shifted toward deferred-transfer strategies.

We agree with Hill *et al*. (2026) that historical Centers for Disease Control and Prevention (CDC) and SART reporting structures incompletely account for delayed transfers and prolonged embryo storage. However, this observation does not invalidate our findings. To the contrary, it reinforces an important point in our article: contemporary IVF practice increasingly promotes repeated treatment cycles and widespread cryopreservation, often resulting in higher patient costs, delayed embryo transfer, and prolonged time to pregnancy. Even if cumulative eventual live birth rates were ultimately unchanged, the patient experience may still reflect reduced treatment efficiency in those terms. These are clinically meaningful outcomes to patients and should not be dismissed merely because delayed-transfer strategies may eventually recover some live births over extended follow-up periods.

Regarding the definition of an ‘add-on’, freeze-all approaches, like many other IVF add-ons, were incorporated into routine clinical practice after 2010 without adequate prior validation through well-designed studies. Therefore, disputing their classification as add-ons is largely semantic and does not alter the underlying reality. And, similar to many other so-called add-ons, freeze-all strategies have also become widely overutilized in contemporary IVF practice.

In their Letter, Hill *et al*. (2026) stated that we concluded that USA IVF efficiency is declining solely because of embryo banking. However, this is not the case. In our article (Gleicher *et al*., 2026), we were very clear that no one ‘add-on’ was singularly responsible for declining cycle outcomes. In contrast, we opined that IVF ‘add-ons’ collectively were likely responsible, with embryo banking representing only one major contributing factor. Other important contributors include PGT-A, routine single embryo transfer, routine blastocyst-stage transfer, and related interventions.

Hill *et al*. (2026) also argue that exclusion of banking cycles restores stable national success rates. Yet this argument raises an obvious methodological question: if contemporary IVF increasingly depends on serial banking cycles before transfer, should those additional cycles truly be excluded from assessments of treatment efficiency? Patients experience those cycles as real treatment burden, cost, delay, and risk.

We also did not, as suggested, create a ‘personal metric’ for cycle efficiency because the question we asked in our article was very simple: What has been happening to live births from autologous IVF cycles after cycle start in the USA over approximately the last 15 years? We did not ask what specifically caused the decline, though we (and others) have for quite some time argued that IVF ‘add-ons’ are primarily responsible for declining IVF cycle outcomes (Gleicher *et al*., 2019).

Hill *et al*. (2026) then also make the rather remarkable statement that, ‘in their opinion’, embryo banking ‘expands treatment options and future fertility potential’. While we appreciate the authors’ established professional expertise, experts’ opinions represent the lowest level of evidence, and it is these that the introduction of IVF ‘add-ons’ since 2010 has largely been based upon. Indeed, the American Society for Reproductive Medicine (ASRM) took until September 2024 to acknowledge that PGT-A does not improve IVF outcomes in general patient populations.

Moreover, there is real evidence that embryo banking not only fails to improve IVF cycle outcomes but, in several ways, especially in poor-prognosis patients, may actually harm cycle outcomes (Orris *et al*., 2010; Kushnir *et al*., 2016; Ge *et al*., 2023).

In our article (Gleicher *et al*., 2026), we did not claim, as Hill *et al*. (2026) suggest, that embryo banking is ‘bad’ for patients. There are, of course, valid reasons for embryo banking. But routine embryo banking can indeed be ‘bad’ for many patients, as any unnecessary routine treatment can be. The mere requirement for an additional freeze-thaw cycle introduces the possibility of embryo loss and therefore reduced cumulative pregnancy chances, even before considering additional treatment costs. This is not a ‘personal viewpoint’, but a fact.

We also disagree with Hill *et al*.’s (2026) argument that, once embryo banking cycles are excluded, IVF efficiency in the USA has remained stable (at ∼32%). First, this was not the question we addressed. More importantly, however, the argument itself is flawed: All IVF ‘add-ons’ introduced since ∼2010 were intended to improve IVF outcomes, not merely to maintain them. Therefore, maintaining similar outcomes despite substantially increased treatment burden and costs already represents a decline in efficiency. As a sidenote, largely because of increasing utilization of IVF ‘add-ons’, IVF cycle costs in the USA almost doubled during the study period after ∼2010 (Gleicher *et al*., 2026).

Furthermore, our study (Gleicher *et al*., 2026) did not claim that embryo banking alone caused declining IVF efficiency, nor did it argue that all cryopreservation is harmful. Rather, our analysis identified a consistent national trend coinciding with major shifts in IVF practice after 2010, including dramatic increases in embryo banking, PGT-A utilization, freeze-all strategies, and other IVF ‘add-ons’. Considering national outcome data, it seems more than reasonable to question whether these changes have ultimately improved cumulative patient-centered outcomes proportional to their increasing complexity, cost, and procedural intensity.

Finally, after introducing themselves in the opening sentence of their letter as leaders of SART, the three authors conclude their critique of our paper by acknowledging important deficiencies in the CDC and SART databases. Because interpretation of national IVF practice trends necessarily relies on these datasets, we hope that they and other SART leaders will appreciate our efforts to draw attention to these limitations and will continue working toward improving registry transparency and outcome reporting systems to ensure accurate and fair representation of contemporary IVF practice.

No panic at all!

## Disclosures

The authors declare no conflicts of interest related to this work.
